# 5-chloro-3-(2-(2,4-dinitrophenyl) hydrazono)indolin-2-one: synthesis, characterization, biochemical and computational screening against SARS-CoV-2

**DOI:** 10.1007/s11696-023-03274-5

**Published:** 2024-03-14

**Authors:** Felicite Majoumo-Mbe, Neba Abongwa Sangbong, Alain Tadjong Tcho, Cyril T. Namba-Nzanguim, Conrad V. Simoben, Donatus B. Eni, Mustafa Alhaji Isa, Adi Narayana Reddy Poli, Joel Cassel, Joseph M. Salvino, Luis J. Montaner, Ian Tietjen, Fidele Ntie-Kang

**Affiliations:** 1 Department of Chemistry, Faculty of Science, University of Buea, P. O. Box 63, Buea, Cameroon; 2 Center for Drug Discovery, Faculty of Science, University of Buea, P. O. Box 63, Buea, Cameroon; 3 Department of Microbiology, Faculty of Sciences, University of Maiduguri, PMB 1069, Maiduguri, Borno State Nigeria; 4 The Wistar Institute, 3601 Spruce Street, Philadelphia, PA 19104 USA; 5 Institute of Pharmacy, Martin-Luther University Halle-Wittenberg, Kurt-Mothes-Strasse 3, 06120 Halle (Saale), Germany

**Keywords:** Angiotensin-converting enzyme 2 receptor, Antivirals, Coronavirus, Isatin hydrazine, Molecular docking, SARS-CoV-2 spike

## Abstract

Chemical prototypes with broad-spectrum antiviral activity are important toward developing new therapies that can act on both existing and emerging viruses. Binding of the SARS-CoV-2 spike protein to the host angiotensin-converting enzyme 2 (ACE2) receptor is required for cellular entry of SARS-CoV-2. Toward identifying new chemical leads that can disrupt this interaction, including in the presence of SARS-CoV-2 adaptive mutations found in variants like omicron that can circumvent vaccine, immune, and therapeutic antibody responses, we synthesized 5-chloro-3-(2-(2,4-dinitrophenyl)hydrazono)indolin-2-one (H_2_L) from the condensation reaction of 5-chloroisatin and 2,4-dinitrophenylhydrazine in good yield. H_2_L was characterised by elemental and spectral (IR, electronic, Mass) analyses. The NMR spectrum of H_2_L indicated a keto–enol tautomerism, with the keto form being more abundant in solution. H_2_L was found to selectively interfere with binding of the SARS-CoV-2 spike receptor-binding domain (RBD) to the host angiotensin-converting enzyme 2 receptor with a 50% inhibitory concentration (IC_50_) of 0.26 μM, compared to an unrelated PD-1/PD-L1 ligand–receptor-binding pair with an IC_50_ of 2.06 μM in vitro (Selectivity index = 7.9). Molecular docking studies revealed that the synthesized ligand preferentially binds within the ACE2 receptor-binding site in a region distinct from where spike mutations in SARS-CoV-2 variants occur. Consistent with these models, H_2_L was able to disrupt ACE2 interactions with the RBDs from beta, delta, lambda, and omicron variants with similar activities. These studies indicate that H_2_L-derived compounds are potential inhibitors of multiple SARS-CoV-2 variants, including those capable of circumventing vaccine and immune responses.

**Supplementary Information:**

The online version contains supplementary material available at 10.1007/s11696-023-03274-5.

## Introduction

**Fig. 1:**
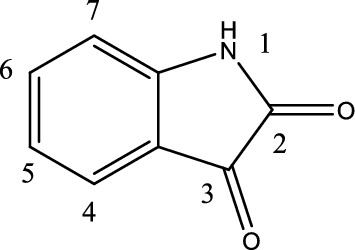
Isatin

The natural product isatin (Fig. 1) serves as a precursor for many bioactive molecules and it is a versatile substrate that can be modified. Isatin derivatives, mostly those substituted at C-3 such as isatin-3-hydrazones, are generally employed as ligands in coordination Chemistry (El-Sawi et al. 2011; Joshi et al. 1980; Snavely and Un 1981; Radovanović and Andelković 1998; Vine et al. 2007). Synthesis of isatin derivatives have gained attention in recent years due to their biological potential as anticancer (Abadi et al. 2006; Vine et al. 2007; Ashraf et al. 2006; Han et al. 2014; Singh et al. 2012; Solomon et al. 2009; Uddin et al. 2007; Vine et al. 2009), antimalarial (Kumar et al. 2014; Raj et al. 2014), antiviral (Abbas et al. 2013; Zhang et al. 2014; Sin et al. 2009), and antimicrobial agents (Kumar et al. 2010; Nandakumar et al. 2010). For example, it has been reported that halogenation at C-5 produces compounds with increased antimicrobial activity (Gurkok et al. 2008; Nathani et al. 2011; Nain et al. 2023; Patel et al. 2006). The in silico evaluation of some isatin-hydrazone derivatives has also been reported and shown to exhibit diverse properties, including potential interactions with topoisomerase, dihydrofolate reductase, and Chikungunya virus envelope and protease proteins, among others (Bittencourt et al. 2016; Mishra et al. 2016; Velasques et al. 2017).

Severe acute respiratory syndrome coronavirus 2 (SARS-CoV-2) causes Coronavirus Disease 2019 (COVID-19), that spread worldwide with major effects on human morbidity and mortality (WHO, 2019). While SARS-CoV-2 has likely become endemic since 2023, it continues to cause substantial mortality worldwide, particularly in high-risk populations such as elderly and immunocompromised individuals. SARS-CoV-2 binds to and infects host cells via its trimeric spike glycoprotein, where the receptor-binding domain (RBD) of the S1 segment can directly interact with the host angiotensin-converting enzyme II (ACE2) receptor to gain cellular entry (Xiu et al. 2020). Antagonism of this RBD-ACE2 interaction, for example by therapeutic antibodies such as REGN10933 (Casirivimab) and REGN10987 (Imdevimab), can inhibit multiple variants of SARS-CoV-2 cellular entry and SARS-CoV-2 infection (Starr et al. 2021). Small molecules that can also disrupt this RBD-ACE2 interface may therefore also be developed into lead compounds to disrupt SARS-CoV-2 infection and mitigate COVID-19 progression.

Computer-aided drug design methodologies do not claim to find lead compounds but could accelerate the process of finding a lead compound (Kontoyianni 2017; Lionta et al. 2014; Baig et al. 2016). These often involve structure-based methods when the molecular structure of the drug target is known, e.g., molecular docking to determine the affinity and orientation of a small molecule within a receptor-binding site (Meek and Weaver 2022), molecular dynamics simulations to test for the stability of a small molecule within a receptor site (Esmaielbeiki et al. 2014; Arcon et al. 2021; Kontoyianni 2017; Rogers et al. 2023) and structure-based pharmacophore methods to assist in screening for putative small molecule binders to a receptor based on how well the three-dimensional (3D) structural features of the small molecules correspond to those of known binders (Baig et al. 2016; Wermuth 2006; Urbina et al. 2022). Ligand-based methods often do not require a knowledge of the structural features of a receptor site (Ferreira et al. 2015; Vazquez et al. 2020). These include quantitative structure–activity relationships (QSAR), ligand-based pharmacophore querying methods, and most recently, artificial intelligence/machine learning (AI/ML) models (Selvaraj et al. 2022; Subramanian et al. 2022; Namba-Nzanguim et al. 2022; Turon et al. 2023). Among these methods, the most cite is molecular docking and scoring techniques have been proven to be efficient ways of identifying active compounds from an electronic library of compounds by a what is commonly called virtual screening (Morris & Lim-Wilby 2008; Chen 2015; Lohning et al 2017). Besides, from the docking orientation of ligand poses, there is often a basis for explaining observed biological activities, mostly in vitro activity concentrations and binding affinities based on the structural interactions between the ligand and the target receptor or the drug target (Kontoyianni 2017; Baig et al. 2016; Rogers et al. 2023).

We recently reported on some new hydrazones with biological activity (Majoumo-Mbe et al. 2015, 2019; Nfor et al. 2013; Yong et al. 2016). Based on preliminary docking studies using the spike RBD from the parental SARS-CoV-2 variant (Wuhan), we hypothesized that hydrazone derivatives may be able to disrupt SARS-CoV-2 spike/ACE2 interactions inclusive of mutations in spike that have arisen in subsequent SARS-CoV-2 variants. Based on this, we now report on the synthesis, characterization, and biochemical and computational screening on SARS-CoV-2 spike of a hydrazone derived from 5-chloroisatin and 2,4-dinitrophenylhydrazine.

## Experimental

### Materials

#### Chemicals

Reagent grade 5-chloroisatin, and 2,4-dinitrophenylhydrazine were purchased from Sigma-Aldrich. Ethanol as solvent and concentrated acetic acid were used as purchased.

#### Physical measurements

Elemental analyses were performed with a Thermo Flash EA-1112 series CHNS-O Elemental Analyzer. The melting points were determined with a Stuart SMP11 instrument in sealed capillary and are uncorrected. Infrared spectra were obtained (KBr 400–4000 cm^−1^) on ALPHA FT-IR Spectrometer from Bruker. UV–visible spectra were carried out with GENESYS 10S UV–Vis spectrophotometer. A Bruker AV 400 MHz Spectrometer was used for the 1H and 13C NMR analysis. Mass spectra were obtained on JEOL Gemate II and Autoflex spectrometers from Bruker.

#### General procedure for synthesis of 5-chloro-3-[2-(2,4-dinitro- phenyl)hydrazono]indolin-2-one (H_2_L)

**Scheme 1:**
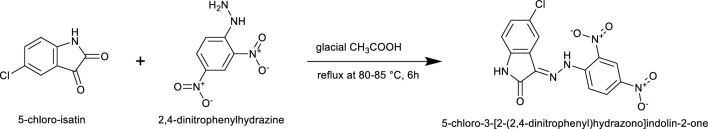
Synthesis of target compound

To a 200-mL ethanolic solution of 5-chloro-isatin (1.5 g, 8.28 mmol) and 2,4-dinitrophenylhydrazine (1.64 g, 8.28 mmol) was added a catalytic amount of concentrated glacial acetic acid (three drops) under reflux at 80–85 °C (see Scheme 1). The resulting solution was further stirred for 6 h. After completion of the reaction, the orange-reddish precipitate obtained after cooling overnight was filtered and washed with methanol (100 mL × 2) and dried. Yield 63%; mp > 350 °C; ^1^H NMR (400 MHz, DMSO-d6) *δ* ppm 11.69 (s, 1H), 11.05 (s, 1H), 8.92 (d, *J* = 2.6 Hz, 1H), 8.61 (dd, *J* = 9.4, 2.6 Hz, 1H), 8.14 (d, *J* = 9.4 Hz, 1H), 7.91 (s, 1H), 8.61 (dd, *J* = 9.4, 2.6 Hz, 1H), 6.98 (d, *J* = 8.3 Hz, 1H). ^13^C NMR (100 MHz, DMSO-d6) *δ* ppm 164.36, 143.99, 143.06, 140.59, 138.08, 133.06, 132.94, 130.99, 126.29, 124.24, 122.98, 117.36, 116.92, 113.07. FTIR (max/cm^−1^): 3372w, 3336w, 3188br, (OH, NH), 3104w, 3057w, 1729m (C=O), 1692m (C=N), 1612s, 1579s, 1497s (NO_2_), 1470s, 1449m, 1338s (NO_2_), 1307s, 1269s, 1230m, 1177s, 1135m, 1109s, 1046m, 847m, 830s, 796m (C–Cl), 719m. Elemental analysis (%): Found: C, 46.40; H, 2.2; N, 19.3 (M+ , 361) C14H8ClN5O5; Calcd (%): C, 46.55; H, 2.2; N, 19.0. UV–vis: max (DMSO/nm) 271, 391, 420sh, 560. ESI (methanol) *m/z* = 362.1 (M+ , 30%), 360.1 (100, M–2H), 307 (5, M–CO,–HCN).

#### AlphaScreen binding assays

AlphaScreen assays were performed as described previously (Tietjen et al. 2021). For RBD-ACE2 assays, 2 nM of ACE2-Fc (Sino Biological, Chesterbrook, PA, USA) was incubated with 5 nM HIS-tagged SARS-CoV-2 Spike-RBDs representing ancestral (“Wild-type” (WT)), beta, delta, lambda, or omicron sequences (SinoBiological) in the presence of 5 μg/mL nickel chelate donor bead in a total of 10 μL of 20 mM Tris (pH 7.4), 150 mM KCl, and 0.05% CHAPS. Test compounds were diluted to 100 × final concentration in DMSO. 5 μL of ACE2-Fc/Protein A acceptor bead was first added to the reaction, followed by 100 nL test compound and then 5 μL of RBD-HIS/Nickel chelate donor beads. All conditions were performed in duplicate. Following incubation at room temperature for 2 h, luminescence signals were measured using a ClarioStar plate reader (BMC Labtech, Cary, NC, USA). Data were then normalised to percent inhibition, where 100% equaled the AlphaScreen signal in the absence of RBD-HIS, and 0% denoted AlphaScreen signal in the presence of both protein and DMSO vehicle control. To measure PD-1-PD-L1 binding, 0.5 nM of human PD-L1-Fc (Sino Biological) was incubated with 5 nM HIS-tagged human PD-1 (Sino Biological) in the presence of 5 μg/mL protein A and 5 μg/mL nickel chelate donor beads in a total volume of 10 μL of 20 mM HEPES (pH 7.4), 150 mM NaCl, and 0.005% Tween. Proteins and test agents were then added, incubated, and analysed as described above.

#### Selection of crystal structure of spike/ACE2 receptor

At the time of this study, four three dimensional (3D) structures of spike/ACE2 complex of SARS-CoV-2 were available from Protein Data Bank (PDB) (Berman et al. 2000; Burley et al. 2017, 2018) and had been solved via X-ray crystallography (PDB codes: 6M0J, 6VW1, 6M17 and 6LZG). The crystal structure 6M0J (Lan et al. 2020) was chosen due to high-resolution and domain completeness. The crystal structure of the Spike RBD/ACE2 complex has 832 amino acid residues divided into two chains (A and E). Chain A is the N-terminal peptidase domain of ACE2 which has 603 residues, while Chain E is the receptor-binding domain of the Spike protein from SARS-CoV-2 and has 229 amino acids residues. The structure also had bound metallic cofactors (Zn^2+^ and Cl^−^), *N*-Acetyl glucosamine (NAG), and water molecules.

#### Molecular docking procedures

Generally, molecular docking procedures were performed using similar methods as reported in our previous published papers (Simoben et al. 2018, 2021; Divsalar et al. 2020).

#### Ligand preparation

The 3D structure of H_2_L was generated using Molecular Operating Environment (MOE, Chemical Computing Group 2017). The ligand was prepared for docking using the LigPrep tool, as implemented in the Schrödinger’s software (Schrödinger 2017), where all possible tautomeric forms were generated. They were subsequently energy-minimised using the integrated Optimised Potentials for Liquid Simulations (OPLS_2005) force field (Banks et al. 2005). Finally, 60 conformers were calculated with ConfGen using the default settings and allowing minimisation of the output conformations (Watts et al. 2010).

#### Protein preparation

The crystal structures of spike/ACE2 complex of SARS-CoV-2 (PDB ID: 6M0J) which is the Wuhan variant, along with the human PD-1/PD_L1 (PDB ID: 4ZQK) were downloaded from the Protein Data Bank (PDB; www.rcsb.org) (Berman et al. 2000; Burley et al. 2017, 2018). All water molecules were deleted using MOE software (Chemical Computing Group 2017). Further preparations of the protein structures preparation were done using the Protein Preparation Wizard of Schrödinger software (Schrödinger 2017; Sastry et al. 2013). At this stage, bond orders were assigned and hydrogen atoms added, missing side chains were filled using PRIME, and the H-bond network was subsequently optimised. The protonation states at pH 7.0 were predicted using the Epik-tool in the Maestro package commercialized by Schrödinger (Schrödinger 2017; Shelley et al. 2007). The structures were finally subjected to a restrained energy minimization step (rmsd of the atom displacement for terminating the minimization was 0.3 Å) using the OPLS2005 force field (Banks et al. 2005). Furthermore, the different variants/mutants of the spike/ACE complex of SARS-CoV were obtained from the Wuhan 6M0J structure (as mentioned above) by mutation (manual replacement of the residues of interest around the spike receptor-binding domain (spike-RBD), using the protein builder module in MOE in the spike protein sequence. Table 1 shows the various mutations carried out on the Wuhan strain or the wild type (WT) spike RBD/ACE2 to derive the various mutants (*β*, *δ*, *λ* and ο).

**Table 1:** Mutations manually carried out on the SARS-CoV-2 Wuhan strain (WT) spike RBD/ACE2 to derive the respective mutants

Beta	Delta	Lambda	Omicron
K417N, E484K, N501Y	L452R, T478K, E484Q	L452Q, F490S	G339D, S371L, S373P, S375F, K417N, N440K, G446S, S477N, T478K, E484A, Q493R, G496S, Q498R, N501Y, Y505H

#### Docking towards the SARS-CoV-2 Spike RBD/ACE2 and the human PD_1/PD_L1

Docking procedures were performed using the Glide program in a similar way as previously demonstrated (Simoben et al. 2018, 2021; Divsalar et al. 2020). In this work three grid boxes for the SARS-CoV-2 viral protein RBD/ACE2 human receptor (PDB ID: 6M0J) and one grid box for the human protein complex PD_1/PD_L1 (PDB ID: 4ZQK, Zak et al. 2015) were generated and using specific protein residues. For the ACE2/SARS-CoV-2 protein (PDB ID: 6M0J), the first grid box of interest constituted of the following amino acid residues D93, Q80, Q68, D277, N272, L125, Y32, K523, F494 and N560 around the ACE2 binding site (further shown and discussed in the Results Section). The second choice was around the spike RBD-ACE2 binding site and was generated using the centroid of the following the residues Q771, Y718, N752, P744, M365, A3769, E05, E311. The last avenue to investigate was where the compounds will preferably bind when the whole structure is explored for the generation of a grid. For this purpose, the following amino acids D597, T598, K516, V321, Q121, K578, A283, S91, N746, Q68, P744, E518 and T610 were used to generate a third grid around the ACE2/SARS-CoV-2 protein. On the other hand, the grid box for the PD_1/PD_L1 structure was generated using the residues F63, V63, N66, Y68, E84, L122, E136, I134 and I126; as reported in literature (Horita et al. 2016; Tang and Kim 2019). For all the generated grid boxes, the sides were set to 36 Å. The generated 3D conformers of the prepared ligand were docked into the different receptor grid files. For the docking process, default settings were used with exception of input ring conformation as well as writing a total of 10 poses per ligand conformer from the 20 poses that were included for each ligand conformer. The GlideScore Standard Precision (SP) mode was used as the scoring function (Halgren et al. 2004).

## Results and discussion

### Synthesis and characterisation of H_2_L

**Fig. 2:**
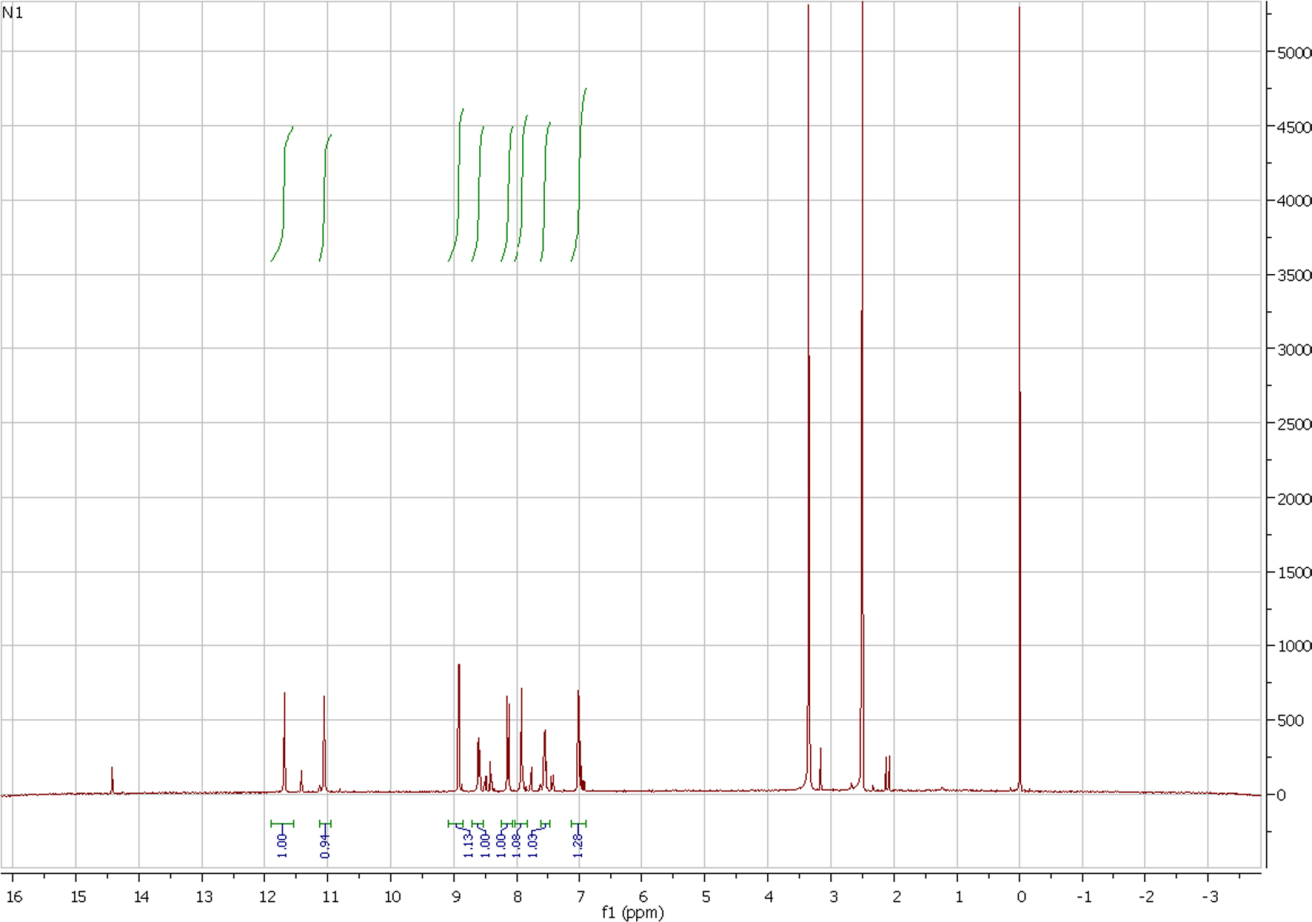
^1^H NMR of H_2_L showing keto–enol forms in DMSO

**Fig. 3:**
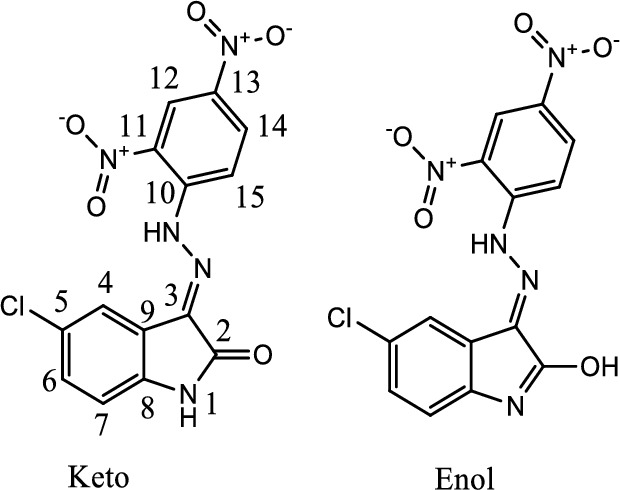
Proposed structure of H_2_L

The ligand H_2_L was synthesized and characterised for biochemical activities. In the NMR spectrum in DMSO (Fig. 2) H_2_L shows trace of the enol form of the ligand (Fig. 3) in solution.

In the ^1^H NMR, the chemical shifts of the N–H groups of the isatin ring and the dinitrophenylhydrazone moieties for the keto form were assigned at 11.05 and 11.7 ppm, respectively, while in the enol form, the OH group of the 5-chloroisatin ring and the N–H group of the dinitrophenylhydrazone moieties were identified at 14.4 and 11.4 ppm, respectively. In the enol form, C12, C9, C7, C5 and C4 signals are downfield shifted with respect to the keto form, while C13 and C15 result in an inverse effect. Amongst the observed signals for both forms, C9 and C4 are the most influenced by the keto–enol equilibrium (shifted about 2.3 and 1.2 ppm, respectively). Similarly, the most affected H-signals are attributed to N–NH, HC15 and HC4 (shifted about 0.3 and 0.2 ppm). The mass spectrum of H_2_L revealed a molecular ions peak at *m/z* 360.1 and 362.1 which is closer to the formula weight (361.7) of the ligand and supported the identity of the proposed structure. In the mass fragmentation of the ligand, a peak corresponding to the loss of CO and HCN can also be observed.

Electronic spectral analysis of H_2_L ligand exhibited three major absorption bands with a shoulder at 271, 391, 420sh and 560 nm. The first observed absorptions can be attributed to the *π*–π* transitions of the aromatic system (Seleem 2011), the *π*–π* transitions of C = O and C = N can be attributed to the second absorption, and the *n*–π* transition due to the lone pairs electron of the oxygen and nitrogen can be attributed to the third absorption. The longest UV-band reflects the charge transfer nature (Seleem et al. 2010, 2011) that gives H_2_L its strong reddish colour. In the IR spectrum of H_2_L, the C=O, C=N and NH vibrations were identified at 1729, 1692, 3336, and 3188 cm^−1^, respectively. Vibrations in the range of 1713–1737, 1685, 3273, and 3193 cm^−1^ were reported for other compounds of isatin hydrazone derivatives with similar environment (Hussein et al. 2019; Jabbar 2018). Additional spectral data are available in the supplementary data (Figs S7–S10).

#### In vitro activities of H_2_L against ligand–receptor interactions

**Fig. 4:**
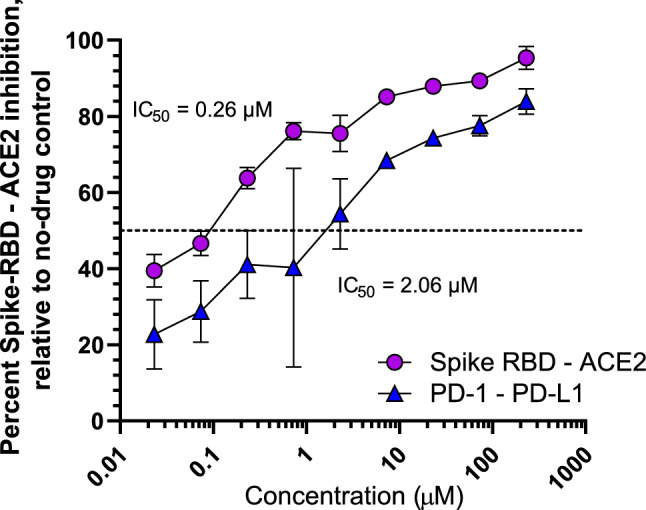
Dose–response curves denoting ability of H_2_L to disrupt luminescence due to SARS-CoV-2 spike RBD–host ACE2 protein-binding (circles) and PD-1-PD-L1-binding (triangles) AlphaScreen assay. Results denote the mean ± S.D. from 3 independent experiments

To determine whether H_2_L may disrupt SARS-CoV-2 entry, we employed a previously-described AlphaScreen technology-based assay (Tietjen et al. 2021; Lan et al. 2020), which uses a SARS-CoV-2 RBD protein containing a C-terminal His tag, bound to an nickel chelate acceptor bead, in addition to a full-length ACE2 peptide with a C-terminal Fc tag bound to a donor protein A bead. When an RBD-ACE2 binding event occurs, the two beads are brought into proximity of each other, at which point excitation at 680 nm results in a singlet oxygen transfer and luminescence at 615 nm. Using this assay, we first asked whether H_2_L could disrupt interactions with RBD from the parental Wuhan variant of SARS-CoV-2, as this sequence provided a useful baseline for understanding H_2_L interactions across multiple variants and was best characterised in this assay (Tietjen et al. 2021). In this approach, we found that H_2_L could disrupt RBD-ACE2 binding with dose dependence and with an IC_50_ of 0.26 μM (Fig. 4; Table 2), in contrast to an IC_50_ of 0.0013 μM for the control therapeutic antibody REGN10933. To assess the selectivity of this interaction, we next determined whether H_2_L could interfere with the unrelated host PD-1/PD-L1 ligand–receptor pair, which we previously observed could be disrupted by the control inhibitor BMS-116611 with an IC_50_ of 0.0040 μM. Using this assay, we also observed dose-dependent inhibition with H_2_L but with a much higher IC_50_ of 2.06 μM (Fig. 4). These results corresponded to a selectivity index [(IC_50_ PD-1-PD-L1)/(IC_50_ RBD-ACE2)] of 7.9 (Table 2), indicating selectivity of H_2_L to disrupt the SARS-CoV-2 RBD-ACE2 interaction. Derivatives of H_2_L with improved cellular tolerance should therefore be assessed for antiviral activity in vitro using pseudovirus-based or replication competent virus-based cellular assays (Tietjen et al. 2021).

**Table 2:** Selectivity studies of binding inhibition of the viral spike RBD-ACE2 protein–protein complex compared to human PD-1-PD-L1 protein–protein complex

Compound	IC_50_ (μM)		Selectivity index
	Spike/ACE2	PD-1/PD-L1	
H_2_L	0.26	2.06	7.9

Results denote the averages from at least 2 independent experiment

#### In silico analysis of H_2_L binding to PD-1-PD-L1 ligand–receptor pair

**Fig. 5:**
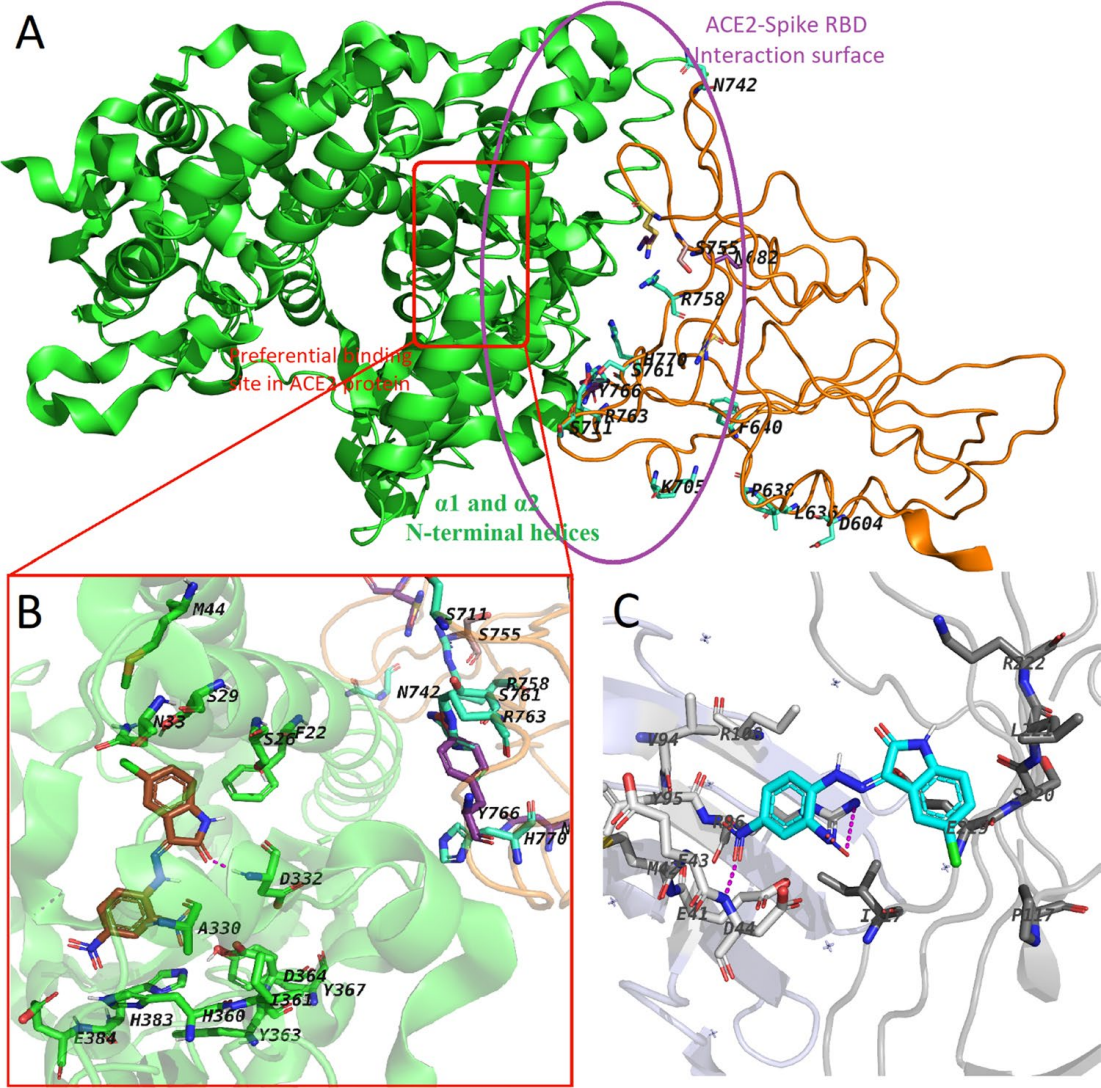
**A** View of the ACE2-Spike RBD complex. The ACE2 protein backbone is shown as green cartoon, while the spike RBD is shown as orange ribbon. Mutation residues are depicted as lincorice-sticks. The ACE2-Spike RBD domain interface and the preferential ACE2 binding site are shown as purple egg sphere and red rectangle, respectively. **B** Close view of the proposed binding mode of the ligand (brown) within the ACE2 binding site. Key residues within the site are shown as green sticks. **C** Proposed binding mode of the ligand (cyan) docked at the interface between PD-1 (light blue) and PD-L1 (grey). For all figures, H-bond interactions are shown as magenta dashed-lines

AlphaScreen showed that the H_2_L ligand was selective towards the inhibition of the spike RBD-ACE2 binding, when compared with PD-1-PD-L1 binding inhibition (Table 2). Interestingly, our work confirmed the comparative studies performed with the unrelated PD-1-PD-L1 ligand–receptor-binding pair. Docking studies showed that, unlike the observed binding of the ligand within the ACE2 binding site for the ACE2-Spike RBD, the ligand was observed to bind between the PD-1 and PD-L1 protein–protein complex as shown in Fig. 5C. The proposed docking pose of the synthesized ligand showed that it had interactions with only two residues (D44 and R96) of the PD-1 surface, thus explaining their non-preference of this complex.

#### In silico analysis of H_2_L binding to RBD-ACE2 ligand–receptor pairs across SARS-CoV-2 variants

To further explain the observed biological activities, computational studies were performed on the spike sequence of the ancestral SARS-CoV-2 variant (i.e., Wuhan variant or “wild-type”, (WT)) as well as SARS-CoV-2 beta, delta, lambda, and omicron variants. Figure 5A depicts the different mutations (as summarized in Table 1 above) that were made to perform this study. The docking studies revealed that the ligand preferentially binds within the ACE2 binding/active site as depicted in Figure S2. This agrees with other studies stipulating that ligands bind within the ACE2 binding site to elicit conformational changes that influence how well the spike RBD would subsequently bind and interact with ACE2 (García-Iriepa et al. 2020; Williams-Noonan et al. 2021). This, therefore, postulates how the synthesised ligand might bind and interact with ACE2 to inhibit the ACE2-spike protein complex formation. Figure 5B exemplifies the binding mode of the ligand within the ACE2 binding for the Wuhan variant. Like the in the other variants, the ligand binds within the ACE2 binding site, interacting with residues on the alpha 1 and alpha 2 (*α*1 and *α*2) N-terminal helices of ACE2, and causing conformational changes on the alpha-(*α*−) and beta-(*β*−) interfaces of the ACE2 protein (García-Iriepa et al. 2020; Williams-Noonan et al. 2021). These conformational changes (as observed in Fig. 5B) are in proximity with the spike RBD. This could imply that the proposed inhibitory mechanism of ACE2 by H_2_L would be expected to occur regardless of sequence changes that occur in the assessed SARS-CoV-2 variants.

#### In vitro activities of H_2_L against RBD-ACE2 ligand–receptor interactions across SARS-CoV-2 variants

Based on these observations, we hypothesized that the mechanism of inhibition of H_2_L was unlikely to be perturbed by mutations that are prevalent in variants of concern. To test this, we repeated the AlphaScreen assays for H_2_L using RBD sequences from beta, delta, lambda, and omicron variants. In these assays, we observed a slightly higher IC_50_ of 447.5 nM for the WT RBD sequence, while no more than a 1.4-fold difference in IC_50_ was observed for any variant (maximum IC_50_ = 628.5 nM using lambda RBD; Table 3). These results for H_2_L agree with the docking studies and suggest that H_2_L derivatives may be useful towards antagonising SARS-CoV-2 entry across multiple variants of concern. In contrast, the control therapeutic antibody REGN10933, while inhibiting WT, delta, and lambda RBD-ACE2 interactions with similar activities (IC_50_s = 0.8–1.4 nM), was ~ 70-fold weaker against the beta RBD (IC_50_ = 90.9 nM) and had no detected activity against the omicron RBD (IC_50_ > 700 nM), consistent with previous reports of fluctuating activity of REGN10933 against SARS-CoV-2 variants (Tietjen et al 2021; VanBlargan et al. 2022).

**Table 3:** Average inhibitory concentrations (IC_50_) of the viral spike RBD/ACE2 binding by the ligand H_2_L for the various strains; Wuhan (WT), beta, delta, delta, lambda and omicron

RBD sequence	IC_50_ (nM)	
	H_2_L	REGN10933
WT	447.5	1.3
Beta	490.3	90.9
Delta	464.1	1.4
Lambda	628.5	0.8
Omicron	614.6	> 700

The docking poses for these variants and towards PD1/PDL1 are available in the Supplementary data as Figs. S1–S6. The therapeutic antibody REGN10933 (Casirivimab) was used as the control. Results are the average of two independent experiments

## Conclusions

We report the synthesis and characterization of a new compound which showed selective antagonism of the binding of the SARS-CoV-2 viral spike protein RBD to the human angiotensin-converting enzyme 2 at sub-micromolar concentrations and across RBD sequences representing multiple SARS-CoV-2 variants of concern including omicron. This activity of H_2_L is consistent with binding of ACE2 leading to subsequent disrupting of protein–protein interactions that are required for RBD binding, and thus presumably SARS-CoV-2 cellular entry and replication. The biological activities revealed that, although the compound was less active than the therapeutic antibody REGN10933 (Casirivimab) for the WT, beta, delta and lambda variants, it was more active against the omicron variant. Besides, for spike/ACE2 binding the reported compound was about eightfold selective when compared with binding to the human PD1/PD-L1 protein complex. Molecular modelling of the interaction between the compound and the angiotensin II binding site of the spike/ACE2 complex reveals interactions with key amino acid residues that could prevent recognition with the RBD of the viral spike. Additionally, the binding against the viral spike/ACE2 complexes of all tested variants showed very similar IC_50_ values, which suggests the design of analogues of H_2_L that could potentially prevent the transmission of the new variants of the SARS-CoV-2 virus. Molecular docking studies also revealed that the synthesized ligand preferentially binds within the ACE2 receptor-binding site in a region distinct from where spike mutations in SARS-CoV-2 variants occur. As H_2_L represents a highly-accessible chemical scaffold that disrupts RBD-ACE2 interactions regardless of SARS-CoV-2 variant sequence and with selectivity over unrelated ligand–receptor interactions such as PD-1-PD-L1, additional studies are therefore warranted to assess H_2_L analogues for their ability to inhibit SARS-CoV-2 variant entry and replication using in vitro cellular infection models. Such leads may be able to support SARS-CoV-2 therapeutic efforts against emerging variants of concern that otherwise circumvent vaccine and host immune responses.

### Supplementary Information

Below is the link to the electronic supplementary material.

Supplementary file1 (DOCX 15304 KB)
