# Supplementary material for: 5-chloro-3-(2-(2,4-dinitrophenyl) hydrazono)indolin-2-one: synthesis, characterization, biochemical and computational screening against SARS-CoV-2
Source: Chem Zvesti. 2024 Mar 14;78(6):3431–41. doi: 10.1007/s11696-023-03274-5 (PMC11055700; doi:10.1007/s11696-023-03274-5)
Supplement: Supplementary file 1 — Supplementary file1 (DOCX 15304 KB) [file 11696_2023_3274_MOESM1_ESM.docx]

**Supplementary Information**

5-chloro-3-(2-(2,4-dinitrophenyl) hydrazono)indolin-2-one: synthesis, characterization, biochemical and computational screening against SARS-CoV-2

**Felicite Majoumo-Mbe^*1^, Neba Abongwa Sangbong^1^, Alain Tadjong Tcho^1^, Cyril T. Namba-Nzanguim^1,2^, Conrad V. Simoben^2^, Donatus B. Eni^1,2^, Mustafa Alhaji Isa^3^, Adi** **Narayana Reddy Poli^4^,** **Joel Cassel^4^, Joseph M. Salvino^4^, Luis J. Montaner^4^, Ian Tietjen^*4^, Fidele Ntie-Kang^*1,2,5^**

^1^*Department of Chemistry, Faculty of Science, University of Buea, P. O. Box 63, Buea.*

^2^*Center for Drug Discovery, Faculty of Science, University of Buea, P. O. Box 63, Buea.*

^3^*Department of Microbiology, Faculty of Sciences, University of Maiduguri, PMB 1069, Borno State, Nigeria.* ^4^*The Wistar Institute, 3601 Spruce Street, Philadelphia, PA 19104 USA* ^5^*Institute of Pharmacy, Martin-Luther University Halle-Wittenberg, Kurt-Mothes-Strasse 3, 06120 Halle (Saale), Germany.*

**^*^**Corresponding author, e-mail: felicite.majoumo@ubuea.cm (FMM); itietjen@wistar.org (IT); fidele.ntie-kang@ubuea.cm (FNK)


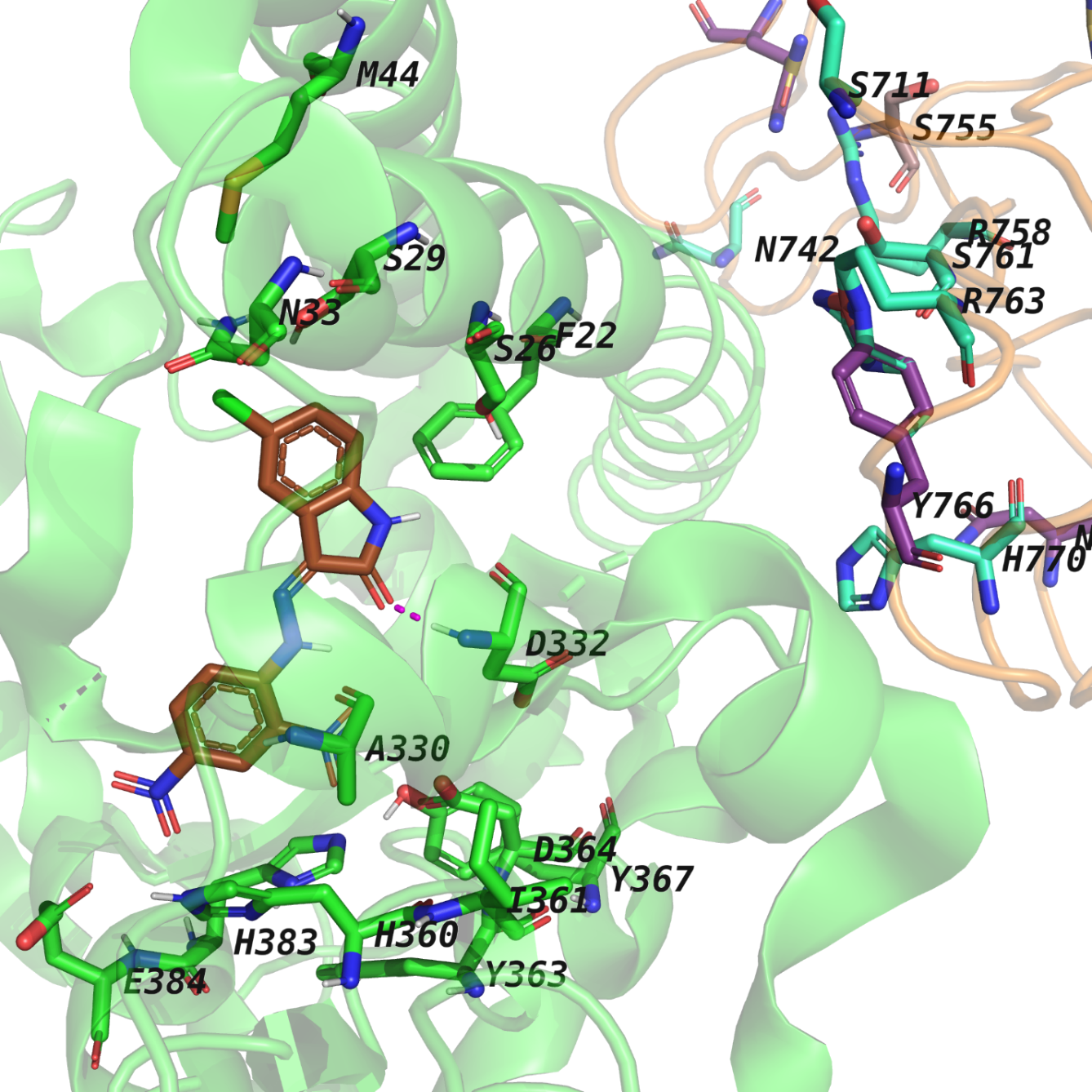


Figure S1: Docking pose of the sythesized ligand (brown stick) within the ACE2 binding site (green cartoon) with the necessary mutations for the Wuhan (WT) variant on the spike RBD (orange ribbon). Visible ACE2 binding site residues are shown as green sticks, while visible residues on spike RBD capable of mutation are shown as sticks.


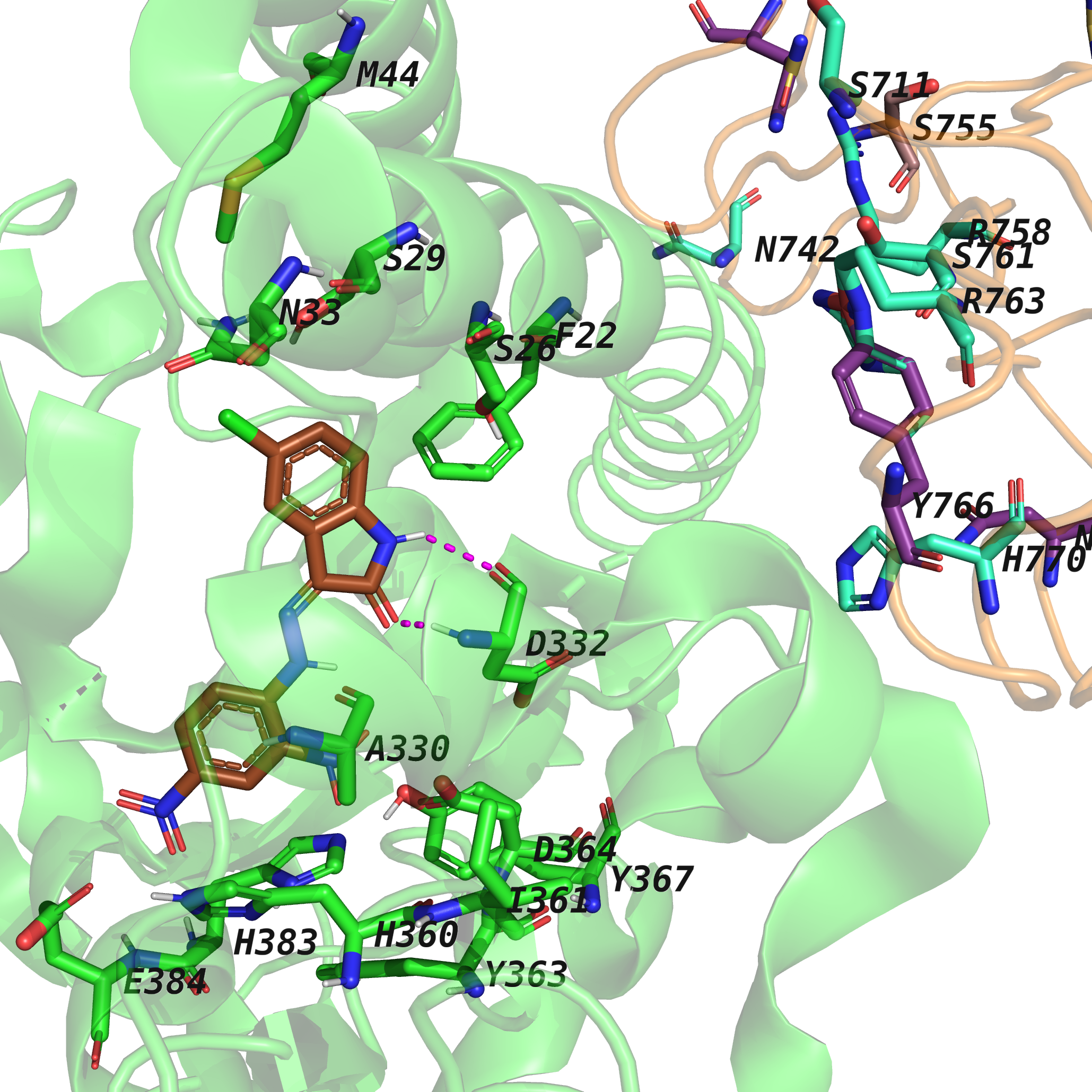


Figure S2: Docking pose of the sythesized ligand (brown stick) within the ACE2 binding site (green cartoon) with the necessary mutations for the beta variant on the spike RBD (orange ribbon). Visible ACE2 binding site residues are shown as green sticks, while visible residues on spike RBD capable of mutation are shown as sticks.


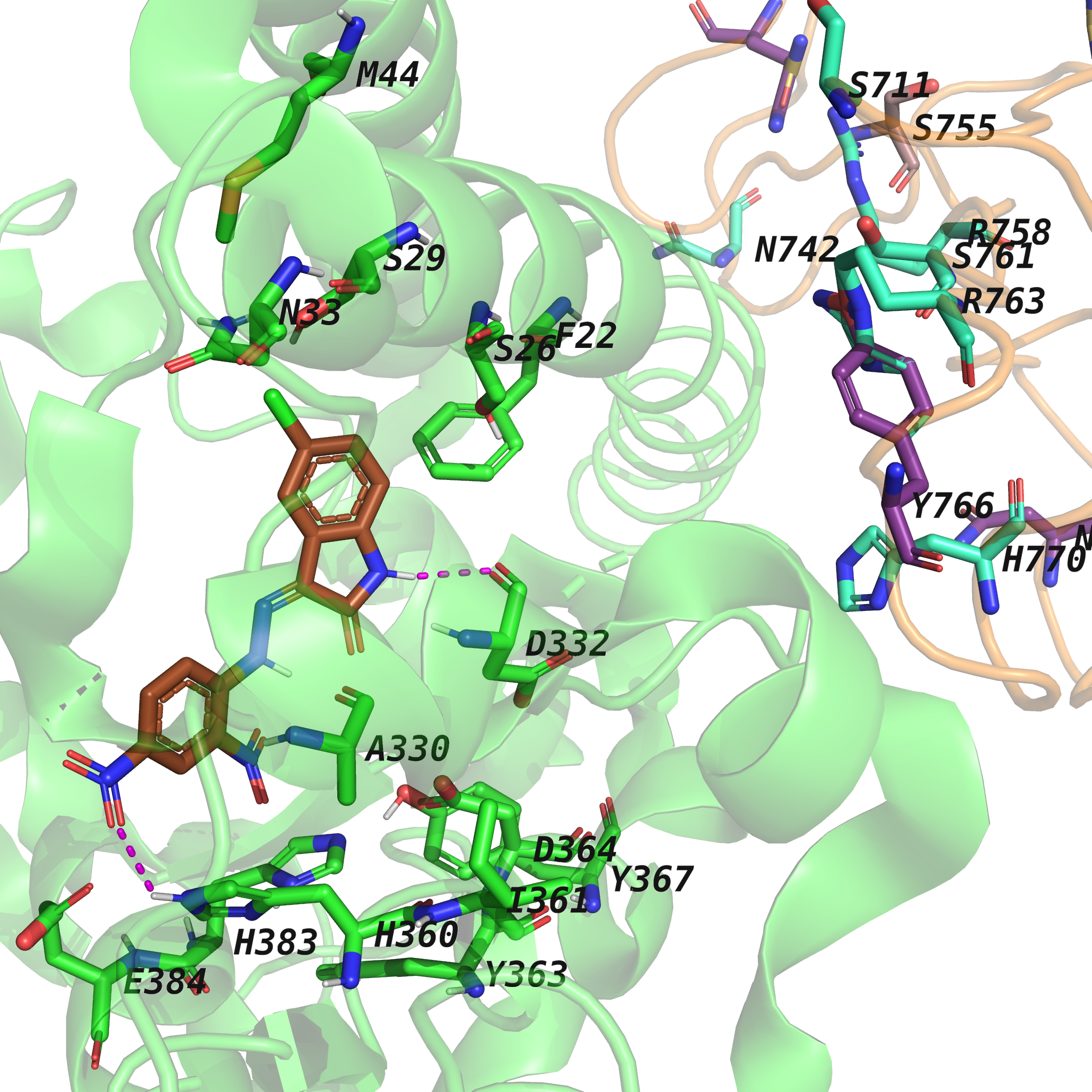


Figure S3: Docking pose of the sythesized ligand (brown stick) within the ACE2 binding site (green cartoon) with the necessary mutations for the delta variant on the spike RBD (orange ribbon). Visible ACE2 binding site residues are shown as green sticks, while visible residues on spike RBD capable of mutation are shown as sticks.


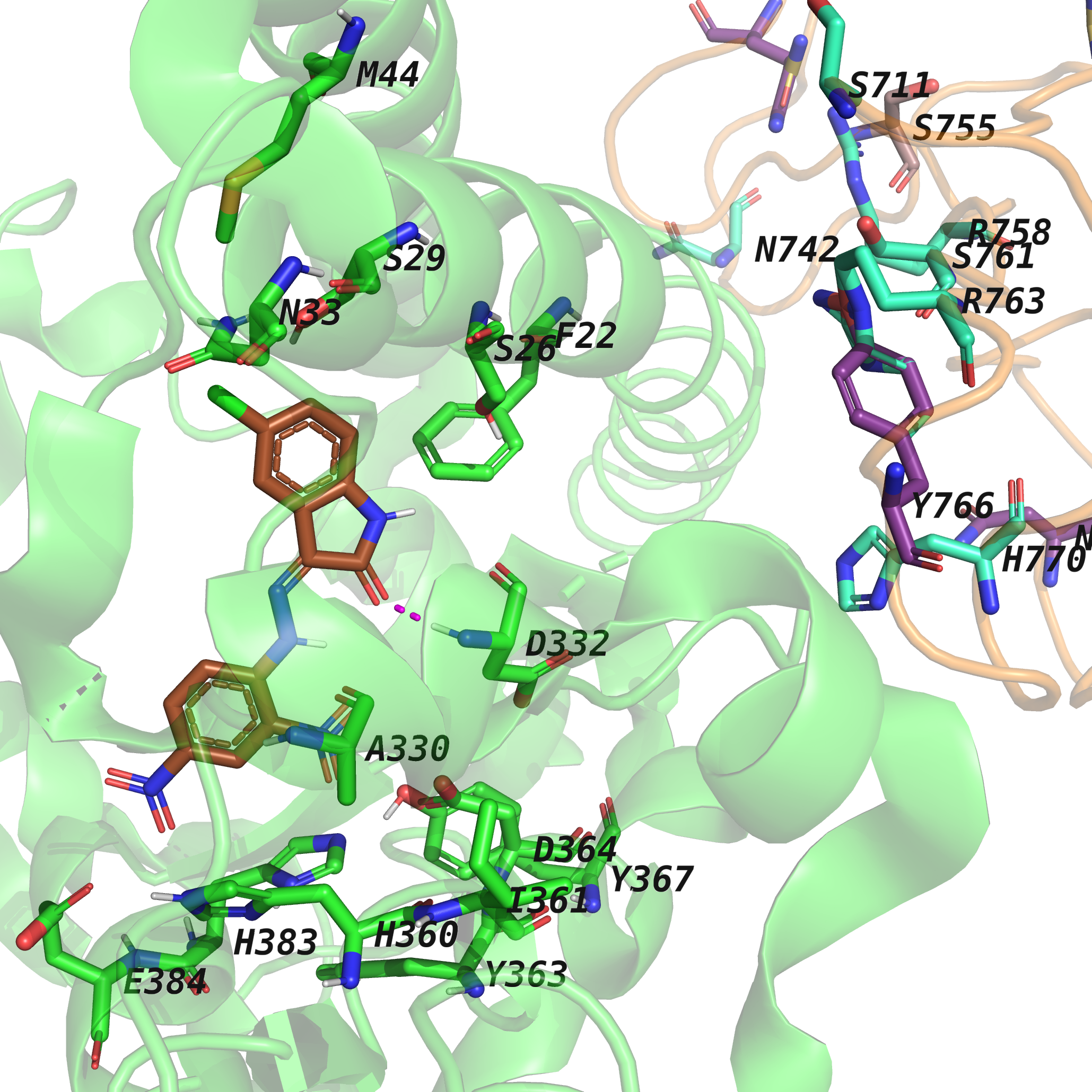


Figure S4: Docking pose of the sythesized ligand (brown stick) within the ACE2 binding site (green cartoon) with the necessary mutations for the lambda variant on the spike RBD (orange ribbon). Visible ACE2 binding site residues are shown as green sticks, while visible residues on spike RBD capable of mutation are shown as sticks.


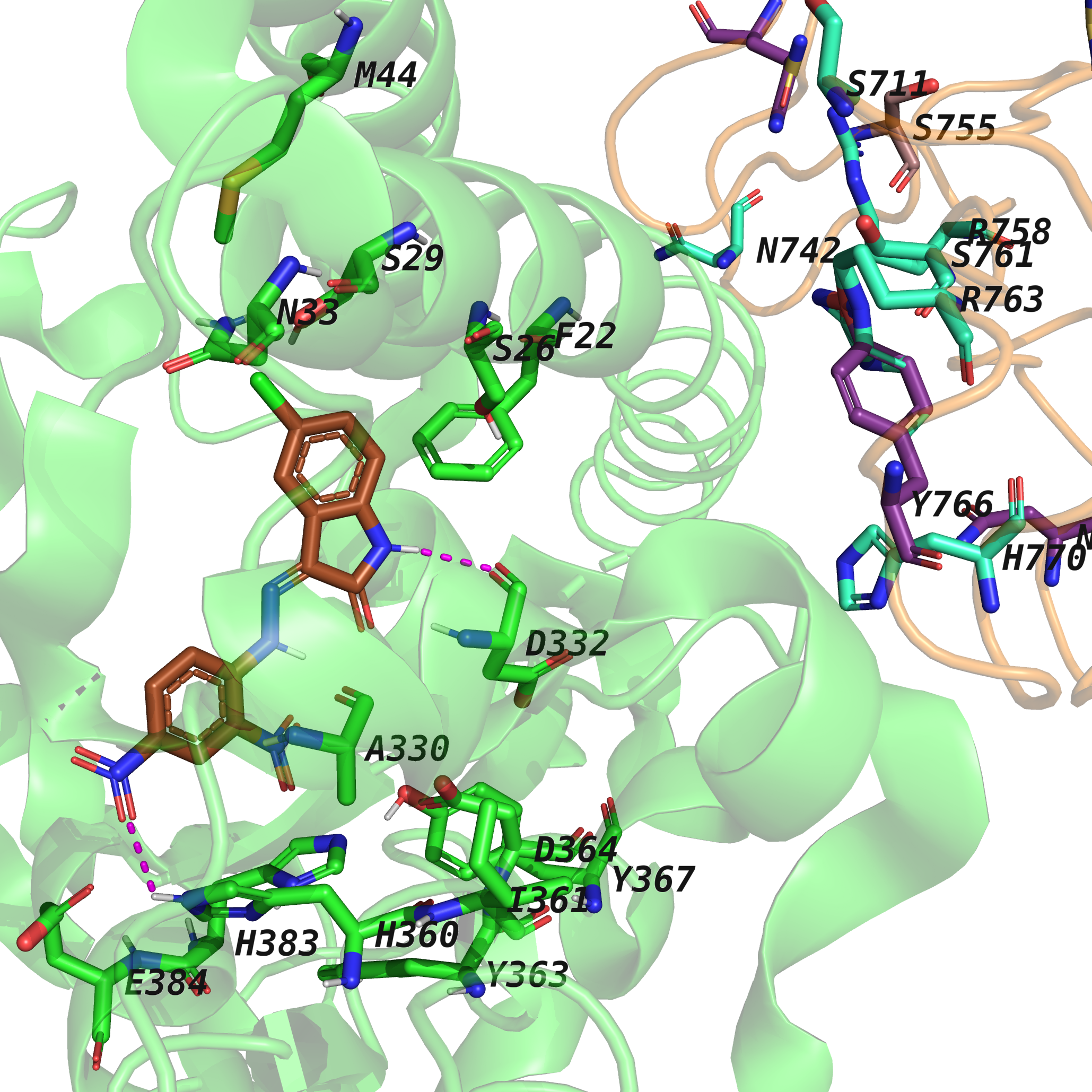


Figure S5: Super Imposition of the pose from all the different variants


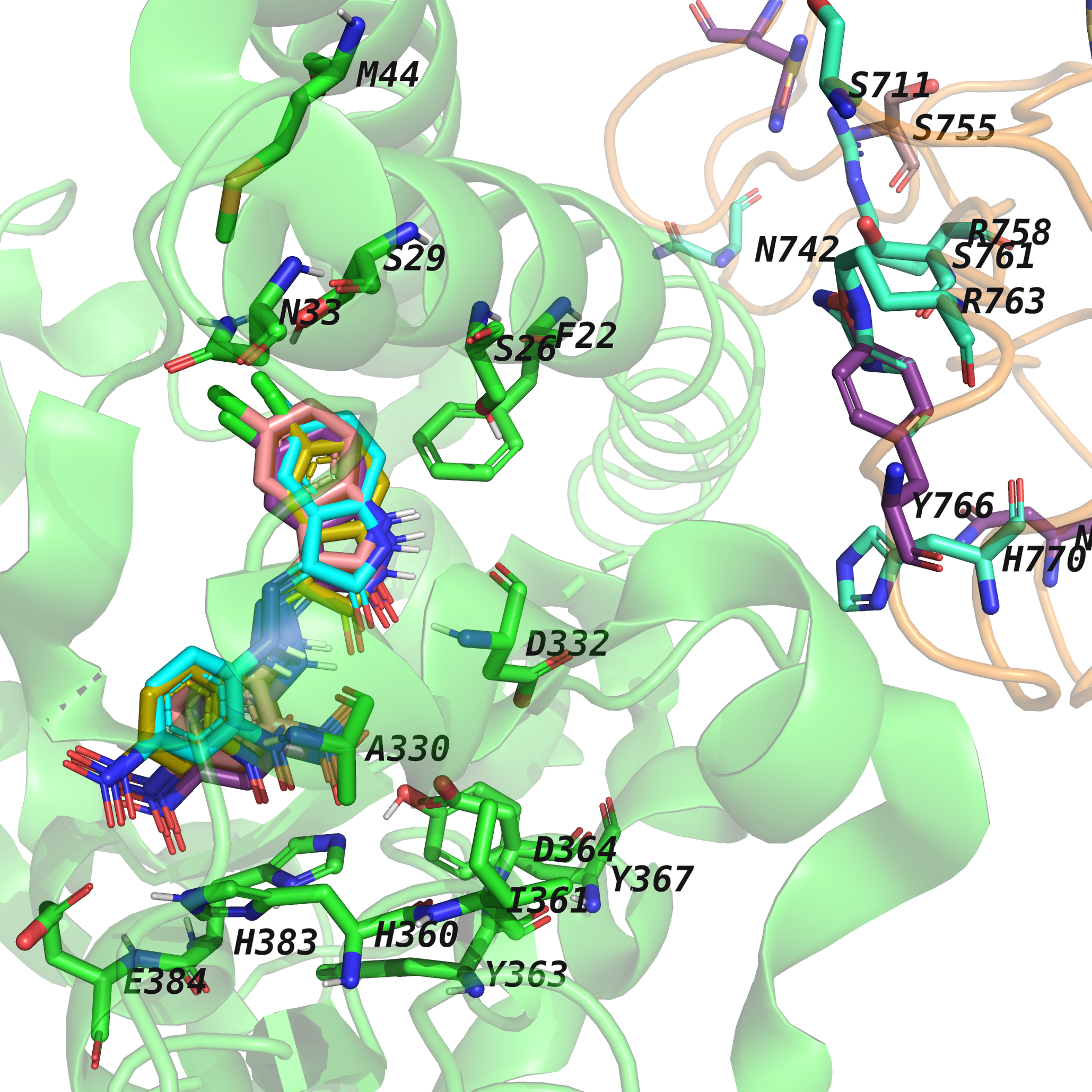


Figure S6: Super imposition of the investigated ligand (brown stick) docking pose from all the different variants within the ACE2 binding site (green cartoon) with the necessary mutations for the Wuhan (WT) variant on the spike RBD (orange ribbon). Visible ACE2 binding site residues are shown as green sticks, while visible residues on spike RBD capable of mutation are shown as sticks.

Fig. S7: LC-Ms data for H_2_L


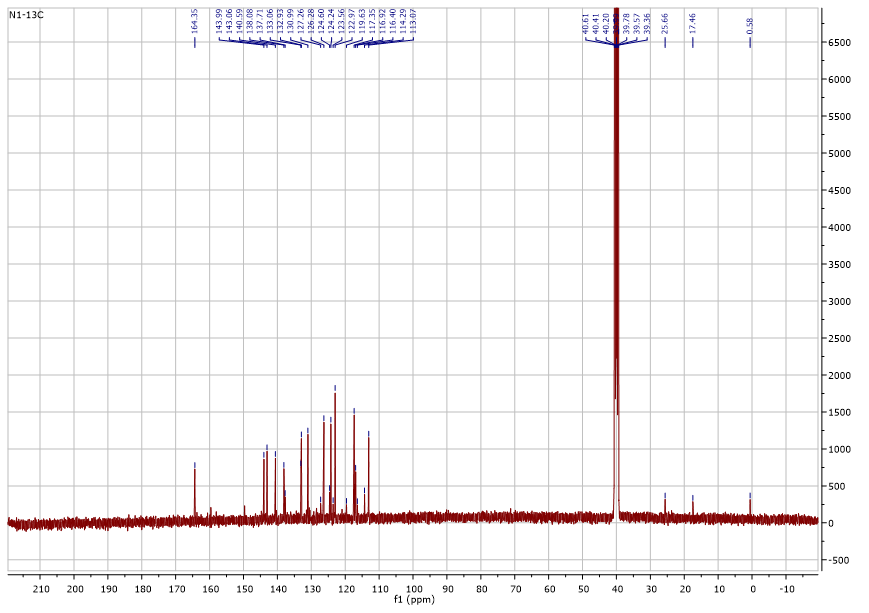

Fig. S8: ^1^H and ^13^C NMR spectra for H_2_L


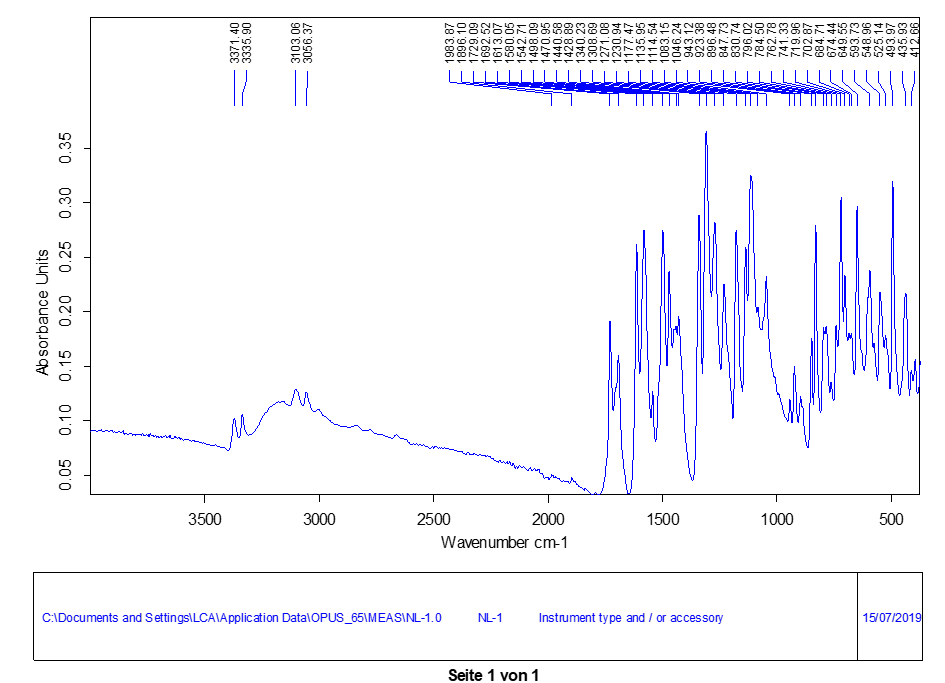


Fig. S9: IR spectrum for H_2_L


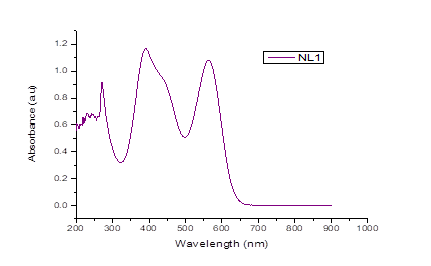


Fig. S10: UV spectrum for H_2_L
